# Influence of Graded Levels of l-Theanine Dietary Supplementation on Growth Performance, Carcass Traits, Meat Quality, Organs Histomorphometry, Blood Chemistry and Immune Response of Broiler Chickens

**DOI:** 10.3390/ijms19020462

**Published:** 2018-02-03

**Authors:** Muhammad Saeed, Xu Yatao, Faiz-ul Hassan, Muhammad Asif Arain, Mohamed E. Abd El-Hack, Ahmed E. Noreldin, Chao Sun

**Affiliations:** 1 College of Animal Science and Technology, Northwest A&F University, Yangling 712100, China; muhammad.saeed@nwafu.edu.cn (M.S.); masif.luawms@gmail.com (X.Y.); 2 Institute of Animal and Dairy Sciences, Faculty of Animal Husbandry, University of Agriculture Faisalabad, Faisalabad 38040, Pakistan; faizabg@gmail.com; 3 Faculty of Veterinary and Animal Sciences, Lasbela University of Agriculture, Water and Marine Sciences, Uthal 90150, Pakistan; asifarain77@nwsuaf.edu.cn; 4 Department of Poultry, Faculty of Agriculture, Zagazig University, Zagazig 44511, Egypt; dr.mohamed.e.abdalhaq@gmail.com; 5 Department of Histology and Cytology, Faculty of Veterinary Medicine, Damanhour University, Damanhour 22516, Egypt; norislam2010@yahoo.com

**Keywords:** blood chemistry, broilers growth, carcass, l-theanine, meat quality

## Abstract

l-theanine is a water-soluble non-proteinous amino acid mainly found in green tea leaves. Despite the availability of abundant literature on green tea, studies on the use of l-theanine as a feed additive in animals, and especially broilers are limited. The objective of this study was, therefore, to evaluate the effect of different dietary levels of l-theanine on meat quality, growth performance, immune response, and blood metabolites in broilers. A total of 400 day-old broiler chicks were randomly divided into four treatment groups using a completely randomized design; C-control, basal diet; 100LT-basal diet + 100 mg l-theanine/kg diet; 200LT-basal diet + 200 mg l-theanine/kg diet; and 300LT-basal diet + 300 mg l-theanine/kg diet. Results revealed that the intermediate level of l-theanine (200 mg/kg diet) showed better results in terms of body weight gain (BWG), feed consumed (FC), and feed conversion ratio (FCR) as compared with the other supplemented groups and the control. The live weight eviscerated weight and gizzard weight were higher in all l-theanine levels as compared to those of the control group. Increased weight (*p* ≤ 0.05) of spleen and bursa were found in group 200LT (200 mg l-theanine/kg diet). Concerning meat color parameters, values for yellowness (b*), and redness (a*) were greater in l-theanine-supplemented groups than the control. Supplementing broiler diet with l-theanine reduced (*p* = 0.02) total serum cholesterol contents while increased HDL. Further analysis revealed lower relative serum cytokines (*IL-2* and *INF-γ*) and reduced mRNA expression of *TNF-α* and *IL-6* in thymus, and *IFN-γ* and *IL-2* in spleen in the treated group. Moreover, supplementation with 200 mg/kg of l-theanine improved antioxidant status in blood by increasing SOD, GSH-Px, and relative CAT levels. It is concluded that the optimum supplementation level of l-theanine is 200 mg/kg of diet because it resulted in improved performance parameters in broilers. However, higher levels of l-theanine (300 mg/kg diet) may have deleterious effects on performance and health of broiler chickens.

## 1. Introduction

Plant compounds that possess antioxidant and antimicrobial properties have the potential to replace antibiotic growth promoters in poultry diets, especially since in most countries, the use of in-feed antibiotics has been banned [1]. The idea behind this intervention is to avoid antimicrobial resistance and residual effects posed by the systematic use of antibiotics, but to maintain high performance, bird health and to produce safe food [2]. Globally, many studies have been conducted to evaluate potential effects of phytochemicals on the performance of birds which revealed beneficial effects on growth, immunity and meat quality [3]. Various parts of medicinal plants and their extracts such as moringa [4], black cumin [5], green tea leaves/flowers [6], quercetin [7], ginger [8], *Yucca schidigera* [9] and yacon [10] have been used in poultry diets as growth promoters.

Green tea (*Camellia sinensis*) is one of the most famous plants; it is used as a traditional drink globally, due to its potential health benefits and biological properties [11]. The dry leaves of green tea contain 92.20% dry matter, 7.80% moisture, 19.32% crude fiber, 82.40% organic matter, 9.80% total minerals, 8.72% ether extract, 36.21% nitrogen-free extract, 18.15% crude protein, and 3002 kcal/kg calculated metabolizable energy [12]. l-theanine is the most abundant amino acid and constitutes about 50% of total amino acids in green tea leaves [13]. l-theanine (γ-Glutamylethylamide) is a unique component of tea causing a catchy aroma taste that helps to remove the bitterness of caffeine and astringency of tea polyphenols [14] and is also responsible for the umami taste of tea [15]. It has potent health benefits like immune boosting, antidepressant- and stress/fatigue-relieving effects as indicated by many studies on humans. Treatment with l-theanine showed a reversal of cognitive impairment, increased secretion of stress hormones and reduced oxidative damage in animals [16]. l-theanine has also been found to increase the level of immunoglobulins in serum (IGg) in mice [17]. In another study [18], it was demonstrated that fruit flies fed with l-theanine were resilient against stress caused by dry and wet starvation in males. Administration of l-theanine at 400 mg kg^−1^ improved immune functions by increasing splenic tissue and decreasing serum corticosterone levels in rats [19,20]. It has also shown a significant antidepressant-like effects in mice [21]. Moreover, the inclusion of l-theanine (400 mg kg^−1^) in diet increased the levels of secretory IgA in the jejunum and serum levels of *IL-2* and *IFN-γ* in chicken [22]. The dietary supplementation of l-theanine in pigs led to improved body weight gain and production of anti-inflammatory cytokines [23]. Many studies, as mentioned above, have been conducted in vitro and in vivo in different species such as cattle, humans, pigs, honeybees and rats but studies on broilers regarding the use of l-theanine are limited. So, this experiment was conducted to evaluate the influence of graded levels of l-theanine on carcass traits, growth parameters, blood metabolites and meat quality characteristics of broilers.

## 2. Results and Discussion

### 2.1. Growth Performance

Significant effects of (*p* ≤ 0.01) dietary l-theanine on growth performance of broilers were observed in this study (Table 1). Dietary supplementation with the intermediate level of l-theanine (200 mg/kg diet) caused an improvement in terms of BWG, FC, and FCR compared to the control and other l-theanine supplemented groups. The improvement of growth parameters due to the supplementation with l-theanine may be attributed to physiological effects of l-theanine like alteration of the microbiome in caeca, but also to its immunomodulatory and antioxidant activities [24].

In the present study, the obvious increase in growth performance of broilers fed with l-theanine (200-mg group) as compared to the control group may be the result of modulation in the mucosal architecture. Our results showed an increase in the dimensions of the intestinal villi by l-theanine supplementation at 200 mg/kg in the diet which seems a most probable factor for the improved performance of birds in this group. However, high levels of l-theanine exhibited adverse effects on these parameters leading to poor performance. Beneficial effects of different dietary levels of green tea and its extract on body weight gain in broilers have been reported earlier [25,26,27]. These studies revealed that lower levels of green tea powder (0.5% vs. 0.75%, 1.0% and 1.5%) and its extract (0.1 g/kg vs. 0.2 g/kg of diet) improved body weight gain, feed intake and carcass traits as compared to control. Contrarily, higher levels of green tea and its extract exerted negatives effects on body weight and feed intake. However, negative effects of green tea supplementation like reduction of body weight (at 1.0 to 1.5% green tea levels) in broilers have also been reported [28]. Insignificant improvement in feed efficiency was observed by Yang et al. [29] as a response of supplementation with graded levels of green tea powder in broiler chickens [30]. Similarly, body weight and feed intake were not significantly affected by green tea supplementation (10 g/kg diet) in broiler chickens [30] This discrepancy may be due to different levels and sources of green tea (i.e., crude powder vs. extracts).

### 2.2. Carcass Traits

Supplementation of l-theanine significantly (*p* ≤ 0.05 or <0.01) improved live weight, eviscerated weight and gizzard weight as compared to the control group, while the other carcass traits were not significantly affected (Table 2). Results revealed that intermediate levels of l-theanine (200 mg/kg diet) resulted in significantly higher eviscerated weight (*p* = 0.010), live weight (*p* = 0.021) and gizzard weight (*p* = 0.003) compared to the control and the other l-theanine supplemented groups. Moreover, this study also showed a significant increase (*p* ≤ 0.05) in weights of bursa and spleen in the group supplemented with 200 mg l-theanine/kg diet (Table 2).

l-theanine has been reported to decrease obesity in mice [31]. While the supplementation of green tea was reported to reduce the abdominal fat in broilers [29].

Several studies have reported the ability of l-theanine to improve immune response [17,32,33,34,35] in humans [36,37], in mice [23] chickens [19] and in rats. The spleen is the organ of T and B lymphocyte aggregation, Li et al. [19] attributed the increase in weight of rat spleen by l-theanine to the increasing differentiation and proliferation of lymphocytes by upregulating *PLC-1 gene* expression. So, the l-theanine has the potential for improving the immune system.

### 2.3. Meat Quality

Effect of l-theanine on meat quality traits including muscle color, pH, and water-holding capacity was investigated (Table 3). Results revealed unique effects of different levels of l-theanine on muscle color. For breast muscle color (BMC), it was observed that L* values decreased (*p* = 0.031) in groups 100LT and 200LT, while it was increased in group 300LT as compared with controls (group C). Values of both a* and b* were increased (*p* ≤ 0.05) in all l-theanine treated groups as compared to controls. For thigh muscle color (TMC), values of L* were decreased (*p* = 0.043); while a* and b* values increased in l-theanine-treated groups as compared to controls. Data of pH of breast and thigh (after 45 min) revealed a significant (*p* ≤ 0.05) decrease in treated groups as compared to controls, which was considered as a good result. Contrarily, water-holding capacity (WHC) increased in both breast (*p* = 0.055) and thigh (*p* = 0.045) muscles in l-theanine-treated groups. In this study, we observed that color of muscles was affected by l-theanine supplementation as a*, L*, and b* values for all treatment groups were in a normal range, however, values of a* and b* were found to be higher in l-theanine groups. It is worth noting that values of meat color are within the acceptable ranges for broiler meat. Our findings are in agreement with Kamath et al. [32] as they reported that green tea extracts increased redness (a*) and yellowness (b*) values of the breast meat. Moreover, our results revealed that muscle tissue color was lighter in meat with a lower pH which is in line with earlier investigations on broilers [32,38]. The color lightness values of poultry meats have been classified as; dark (L* < 46), normal (48 < L* < 51) and pale (L* > 53). Earlier studies assume that pale meat had lower values of pH and a* than normal and dark meat in addition to higher b* values [38,39]. It is generally presumed that chlorophyll, catechins and polyphenolic contents of green tea are responsible for antioxidant activities and color of green tea that may contribute to lighter and dark meat colors [27]. The protective effects of green tea against lipid oxidation and protein oxidative degradation is related to deactivation of hypervalent iron pigments by green tea catechins alone or in combination with other reducing constituents of green tea such as l-theanine [40]. The values of pH of meat from broilers fed l-theanine-enriched diets showed a lesser decrease in WHC of meats in the current study. However, Young et al. [41] demonstrated that lower overall final pH did not cause an overall depression in WHC.

### 2.4. Mucosal Histomorphometry of Small Intestine

**Figure 1:**
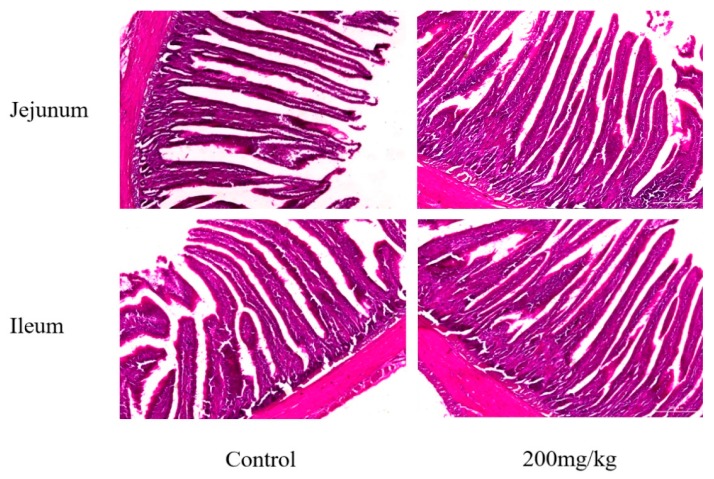
Light micrograph of HE staining showing difference in length of the intestinal villi and crypts in both jejunum and ileum by two treatments, control and 200 mg/kg l-theanine. Scale bar: 200 μm.

Mucosal histomorphometric studies revealed higher villus height (VH) and crypt depth (CD) in the jejunum and ileum of group C (l-theanine@200 mg/kg) especially significantly higher VH in the ileum (*p* ≤ 0.05) compared to control (Table 4, Figure 1). Moreover, higher VH:CD ratio was observed in the jejunum and ileum of this group.

l-theanine promotes gut development and absorption of amino acids in the small intestine by upregulating expression of intestinal gene transporters responsible for the neutral, acidic and basic amino acid absorption [42]. We observed an improvement in intestinal histomorphometry in group 200LT in the jejunum and ileum as compared to the control group. These results suggested that digesta containing l-theanine had better effects on ileum as compared to the jejunum, which might be attributed to higher absorption of l-theanine in the ileum than jejunum that ultimately leads to higher absorption of amino acids. It is generally presumed that the ilium is reported to have better digestibility than duodenum and jejunum in broilers, which improves the performance of the birds [43]. Similar to our results, Mosleh et al. [44] observed a higher level that was significant from a statistical standpoint.

The modulation of gut architecture using different feed additives like organic acids, phytochemicals and probiotics, has been associated with an increase in overall intestinal surface area, villi height and crypts that ultimately enhance nutrient absorption and digestive action of the gut [45,46]. l-theanine has the ability to positively affect mucosal tissue development and even regeneration of atrophied intestinal tissue in rats [28]. The small intestine is the main site for nutrient absorption in which mucosal cells take up available nutrients through their cell membranes and drain into blood circulation. The manipulation of the architecture of the small intestine is presumed to influence nutrient absorption ultimately leading to change in performance. Our results showed an improvement in morphometric parameters of small intestinal-like VH, CD, and VH: CD in the l-theanine-treated (200 mg/kg) group is attributed to enhanced turnover of epithelial cells [31]. Li et al. [19] reported improvement of immune system functions in the rat by l-theanine (400 mg/kg BW) by altering Th2/Th1 cytokine balance, raising 5-hydroxytryptamine and domain in the brain, lowering the serum corticosterone level, decreasing phospholipase *C-1* (*PLC*-1) and increasing *PLC-1* in the spleen. While Wen et al. [22] observed an increase of the levels of IL-2 in the serum and the level of secretory IgA in the jejunum by supplementing l-theanine (400 mg/kg) in the chicken diet.

### 2.5. Histomorphometry of Pectoral and Gastrocnemius Muscles

**Figure 2:**
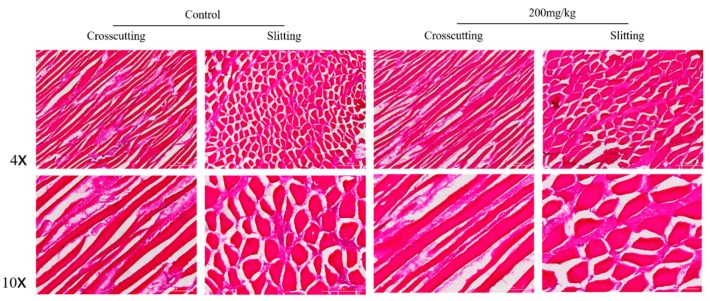
Light micrograph of HE staining showing the gastrocnemius muscle in which the mean fiber cross-sectional area has a wider diameter in 200 mg/kg l-theanine than in controls. Scale bar: 200 μm.

**Figure 3:**
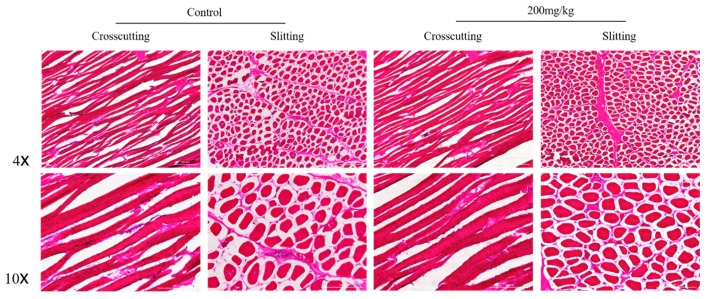
Light micrograph of HE staining showing the pectoral muscle in which there is a similarity in mean fiber cross-sectional area between the 200 mg/kg l-theanine and controls. Scale bar: 200 μm.

The mean fiber cross-sectional area of gastrocnemius muscle was improved in the supplemented group owing to the ability of l-theanine to inhibit production of reactive oxygen species (ROS) from neutrophils aggregated in skeletal muscles (Table 5, Figure 2 and Figure 3). Neutrophils can damage skeletal muscles exhausted by intense exercise. Therefore, l-theanine lowers further gathering of neutrophils and, hence, suppresses exaggerated inflammatory responses and breakdown of skeletal muscle [47,48]. The behavior of bird movements could be the explanation of the increase of thigh muscle weight by Japanese green tea powder feeding [25].

### 2.6. Effect on Blood Chemistry

Dietary l-theanine exhibited non-significant effects on blood parameters except total cholesterol of broilers in this study (Table 6). The supplementation of broiler diet with l-theanine decreased (*p* = 0.02) serum total cholesterol. The intermediate level of l-theanine (200 mg/kg diet) resulted in the lowest total cholesterol levels (1.53 mmol/L) compared to control and other l-theanine levels. Albeit not significant, supplementing broiler diets with l-theanine (100 or 200 mg/kg diet) increased serum HDL (good cholesterol) and reduced LDL (bad cholesterol) contents. Contrarily, a higher level of l-theanine (300 mg/kg diet) exhibited opposite effects (lower HDL and higher LDL-cholesterol levels). Our findings that l-theanine contents of green tea have an ability to lower cholesterol in the blood are in agreement with earlier reports as supplementation of broiler diet with green tea powder or its extract decreased total plasma lipids while increasing HDL [13,18,31,49]. A similar observation has also been recorded in growing Japanese quail [50], rats [51] and mice [33,52].

Conversely, El-Deek and Al-Harthi [53] observed non-significant effects of supplementing green tea in broiler diets (0.5% of diet) on blood cholesterol, which might be due to lower levels of green tea used in this study. Moreover, Xu et al. [54] also observed that addition of Chinese dark tea showed no effect on HDL and serum total cholesterol levels in laying hen. 

The decline in serum cholesterol reported in the above studies might be due to catechins contents of green tea which can lower synthesis of cholesterol [6,55,56] in the body and inhibit absorption of lipids by the intestine [57]. Moreover, l-theanine has also been found to decrease accumulation of lipids in mouse livers leading to improved liver functions [33]. Synthesis of bile salts from cholesterol in liver is the main pathway of elimination of cholesterol from the body, which increases in case of efficient liver functioning, thus leading to reduced serum cholesterol content [25]. So, active liver converts more cholesterol to bile salts which might be another explanation for the decrease of cholesterol in the body.

Neutrophils can damage skeletal muscles exhausted by intense exercise. Therefore, l-theanine lowers further gathering of neutrophils and, hence, suppresses exaggerated inflammatory response and breakdown of skeletal muscle [48]. The behavior of bird movements could be the explanation of the increase of thigh muscle weight by Japanese green tea powder feeding [25].

### 2.7. Immune Response

**Figure 4:**
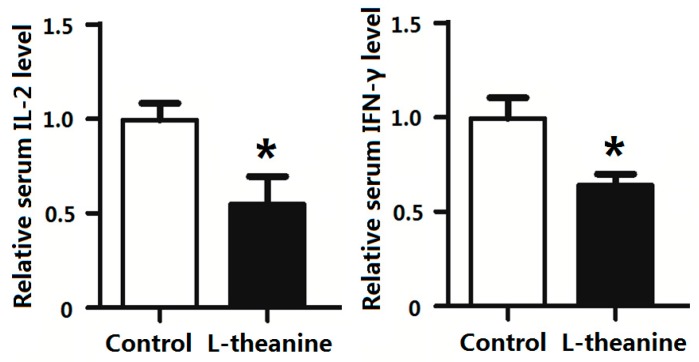
Effects of l-theanine on the relative expression of IL-2 and IFN-γ. Note: Values are means ± SEM. vs. control group, * *p* ≤ 0.05.

**Figure 5:**
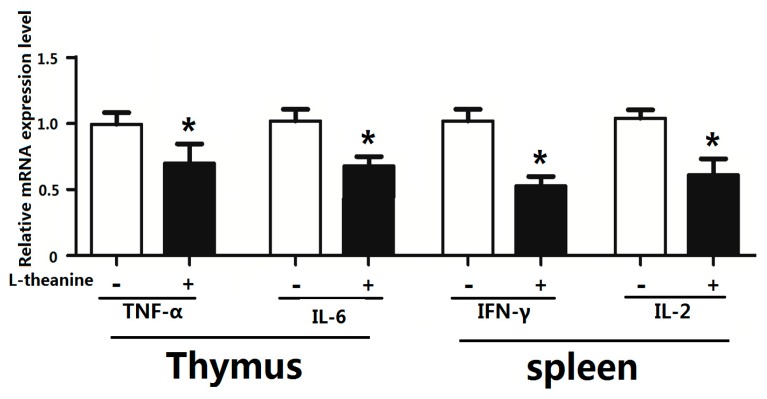
Effects of l-theanine on relative mRNA expression of cytokines in immune tissue. Note: Values are means ± SEM. vs. control group, * *p* ≤ 0.05.

**Figure 6:**
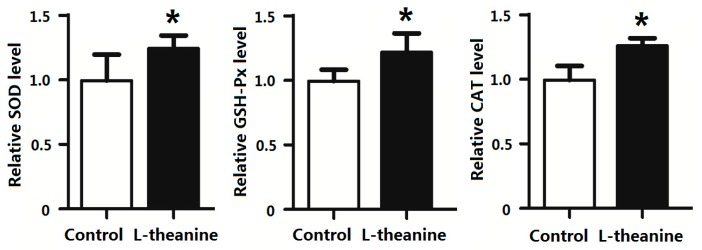
Effect of l-theanine on relative levels of anti-oxidant enzymes status in blood. Note: Values are means ± SEM. vs. control group, * *p* ≤ 0.05.

Pro-inflammatory cytokines mediate immune responses in chicken as they pose potential effects on systemic inflammatory status. The imbalance in cytokine production especially disturbed pro-inflammatory (Th1) and anti-inflammatory cytokines (Th2) ratio results in pathological disorders [58]. In the current study, dietary supplementation of 200 g/kg l-theanine significantly decreased relative serum levels of the pro-inflammatory cytokines *IL-2* and *INF-γ* in broilers (Figure 4). Similarly, l-theanine also lowered mRNA expression of *TNF-α* and *IL-6* in thymus and *IFN-γ* and *IL-2* in spleen (Figure 5). The down regulation of these cytokines (*Th1*) by l-theanine results in better immune homeostasis and prevents further activation of inflammatory responses. Moreover, treatment with 200 mg/kg improved antioxidant status in intestinal tissue by increasing SOD, GSH-Px and relative CAT levels in our study (Figure 6). Improvement in immune function in broilers was observed in response to l-theanine treatment by decreased levels of pro-inflammatory cytokines (*IL-2* and *INF-γ*) and mRNA expression of *TNF-α* and *IL-6* in thymus and *IFN-γ* and *IL-2* in spleen which has been also reported earlier [19]. A significant improvement of immune functions was observed in the rat in response to treatment with l-theanine (400 mg/kg BW). Li et al. [19] explained that l-theanine modulated the immune response by altering serum cytokine balance (*Th2*/*Th1*), elevating 5-hydroxytryptamine and dopamine levels in the brain, lowering serum corticosterone levels and regulating phospholipase *C-1* (*PLC-1*) in spleen. However, Shirui and Xi observed an increase in levels of *IL-2* and *IFN-γ* in serum and level of secretory IgA in the jejunum by supplementing l-theanine (400 mg/kg) in yellow feathered broiler diet. 

Inclusion of l-theanine (200 mg/kg BW) in rat diet increased the status of antioxidant enzymes, decreased inflammatory cytokines and showed protection against PCB-induced oxidative damage in rat brain [22]. Moreover, earlier reports revealed that l-theanine significantly increased activities of anti-oxidant enzymes (SOD, CAT, GSH), thus inhibiting the decrease in antioxidant activity in vitro and in vivo [59]. It has also been observed to reduce oxidative damage in rats by increasing endogenous antioxidant capability. l-theanine has also been reported to reduce CCl4-induced liver damage in rats by decreasing levels of inflammatory cytokines (e.g., *TNF-α* and *IL-β*) while reducing apoptosis [60]. 

## 3. Materials and Methods

### 3.1. Ethical Statement

All procedures and experiments used in this study comply with guidelines of the local institutional ethical committee of the Northwest A&F University (Shaanxi Province, Xianyang, China) regarding care and welfare of animals under study (T/NWSUAF, 235-2014, 2014-05-09).

### 3.2. Design and Feeding Management

A total of 400 day-old broiler chicks (Arbor Acre) were bought from a local hatchery and divided into 4 treatments groups (C, 100LT, 200LT and 300LT). Every treatment had five replicates and contained 20 birds per replicate. Fresh drinking water and feed were provided ad libitum to all birds during the entire experimental period. The total rearing period of six weeks was sub-divided into two phases: a starter phase (1–3 weeks) and finisher phase (3–6 weeks). l-theanine was purchased from a commercial company (Jiangsu Qiang Sheng Chemical Co., Shanghai, China with 98% purity) and was mixed in feed mill with different concentrations at 0, 100, 200 and 300 mg/kg feed for the C, 100LT, 200LT and 300LT treatments groups, respectively, and even did not change in pH value of diets. Basal diets were formulated according to NRC recommendations and were iso-caloric and iso-nitrogenous (NRC, Rockville, MD, USA, 1994) as shown in Table 7. Chicks were housed in a room having 20 pens of uniform size (3 × 4). Pens were whitewashed and disinfected with Bromogeramine (1%) before the start of the experiment. For each pen, wood shaving (layer of 3 inches) was used as litter. Ambient temperature was set 32 °C at placement, and then decreased gradually to achieve 24 °C from week 3 onwards. Lighting was constant on the first day, and then changed from day 2 to the end of the study trial to a regime 21 L:3D.Chicks were vaccinated against Infectious Bursal Disease (IBD) at the age of 8th, 18th and 28th days while for Newcastle Disease (ND) at the age of 5th and 18th days.

### 3.3. Data Collection

During the experiment, weekly and initial body weight per replicate were recorded to estimate weekly body weight acquisition. Feed refusal was also measured for each group during the trial. The recorded data for feed intake and weight gain were utilized to calculate weekly feed consumed (FC) and feed conversion ratio (FCR).

### 3.4. Carcass Traits

For carcass traits, five birds were randomly selected from each group as a bird from each replicate (total = 20 birds). After slaughtering, weights of breast muscle, leg muscle, liver, gizzard, spleen, heart, thymus and bursa were recorded and expressed as g/kg of slaughter weight (SW). Dressed weight of each carcass examined was also calculated (dressed weight = weight of carcass plus the weight of giblets/pre-slaughter weight).

### 3.5. Meat Quality Assessment

The pH values of breast and leg meat samples were determined by immersing a digital pH meter (Hanna, Italy) into meat samples. Meat colors were determined according to yellowness (b*), redness (a*), and lightness (L*) values using an instrument known as Konica Minolta colorimeter CR-400 (Chiyoda-ku, Japan) as demonstrated by Pi et al. [61]. From each sample, connective tissue and visible fat were eliminated. Promptly after measuring meat color and pH, all samples were bundled in polyethylene bags and were kept at −20 °C. After that, all frozen samples were left to thaw at 4 °C in a refrigerator for 12 h. The thawed samples were preserved at 4 °C till further analysis. 

The water-holding capacity was determined by a method reported by Li [19]. The raw meat samples (5 g) were placed among pre-weighed filter papers (18 Whatman No. 1) and pressed for 5 min under 343 N force using a compression machine (SZLH508, ZCME Co., Ltd., Shanghai, China). The pressing loss was calculated as a portion of weight loss before and after compression and expressed as follows:

% Expressible water = (initial weight of the sample − final weight of the sample)/initial weight of the sample × 100.

### 3.6. Immune Response and Antioxidant Status

Growth data revealed better performance of broilers at an l-theanine level of 200 g/kg of diet, so further analysis of immune response was carried out by using samples from this group. For determination of immune response, relative levels of serum cytokines *IL-2* and *INF-γ* were determined using ELISA kits according to manufacturer’s instructions (Cusabio, Wuhan, China). Similarly, mRNA expression levels of *TNF-α* and *IL-6* in thymus and *IFN-γ* and *IL-2* in spleen were determined by the quantitative RT-PCR method as described by [62].

Total RNA form thymus and spleen were extracted with a TRIpure Reagent kit (Takara, Dalian, China) and 500 ng of total RNA was reverse transcribed using the M-MLV reverse transcriptase kit (Takara, Dalian, China). Primers for *TNF-α* and *IL-6 IFN-γ* and *IL-2* were synthesized by Shanghai Sangon Ltd. (Shanghai, China). Quantitative PCR was performed in 25 μL reactions containing specific primers and SYBR Premix EX Taq (Takara, Dalian, China). The levels of mRNAs were normalized to β-actin. The expressions of genes were analyzed by the method of 2^−△△*C*t^ [62]. 

All primers and probes used are presented in Table 8. Moreover, antioxidant status of blood was determined by measuring the levels of superoxide dismutase (SOD), glutathione peroxidase (GSH-Px) and relative catalase (CAT) enzymes in the homogenate by using respective diagnostic kits (Nanjing Jiancheng Bioengineering Institute, Nanjing, China) according to the instructions of the manufacturer. The concentrations of these enzymes were expressed as unit per mg of mucosal protein. 

### 3.7. Blood Chemistry

At the end of the trial, blood samples (5 mL each) from subcutaneous veins were collected in anticoagulant labeled vacutainers from 12 birds per treatment. These samples were used to determine serum parameters, such as triglycerides, total cholesterol, glucose, low-density lipoprotein cholesterol (LDL-C), high-density lipoprotein cholesterol (HDL-C), alanine aminotransferase (ALT) and aspartate aminotransferase (AST) using commercial kits (DDS^®^ Spectrophotometric Kits, Diasis Diagnostic Systems Co., İstanbul, Turkey) with an autoanalyzer Vitros 5.1FS. The collection of blood sera was followed by centrifugation at 3000× *g* for 10 min at 20 °C, and then samples were preserved at −20 °C until laboratory analyses. 

### 3.8. The Histomorphometry of Small Intestinal Mucosa

For histomorphometry, samples (five samples per treatment) of one cm in length segments were sliced from the ileum (three cm proximal to the ileocecal junction) and jejunum (three cm proximal to the Meckel’s diverticulum). Paraffin embedding technique was carried out for samples processing, paraffin blocks were sectioned at 4 μm, and stained with H&E (Haematoxylin and eosin) dyes [63].

From each intestinal segment, three sections were used (one section from serial ten sections). From every section, five complete villi possessing perfect orientation and intact lamina propria were selected indiscriminately for inspection. Therefore, an average of fifteen values were tested for each intestinal sample. 

Slides were examined under a light microscope (at 4× magnification), supported with a digital camera (Leica EC3, Leica, Germany). Images were analyzed by an image processing system photo analyzer (Image J; v1.46r, NIH, Bethesda, MD, USA) as described by [64]. The variables calculated for histomorphological modulations were crypt depth (CD), villus height (VH), villus height to crypts depth ratio (VH: CD) according to [65] and Kiczorowska et al. [66]. 

### 3.9. The Histomorphometry of Pectoral and Gastrocnemius Muscles

Cross and longitudinal dissections of pectoral and gastrocnemius muscle were processed, sectioned and stained for quantification of mean fiber cross-sectional area as previously described [67]. Light photomicrographs at 10× were taken using a Leica light microscope and images were analyzed using Image J.

### 3.10. Statistical Analysis

Data were analyzed for a completely randomized design utilizing GLM procedures of SPSS software version 17.0 (SPSS, Chicago, IL, USA). Following statistical model was used:

Y*_ij_* = μ + T*_i_* + e*_ij_*

where Y*_ij_* = an observation, μ = the overall mean, T*_i_* = effect of l-theanine level (*i* = 0, 100, 200, 300 mg/kg diet) and e*_ij_* = random error. Differences among means were calculated by using post-hoc Newman-Keuls. 

## 4. Conclusions and Application

(1) l-theanine is an amino acid (the active component of green tea) which could be utilized as a natural feed additive to replace antibiotics to improve the overall performance of broilers.
(2) l-theanine showed promising effects on the immune response in broilers by decreasing pro-inflammatory cytokines and enhancing the anti-oxidant capacity of the body by increasing levels of antioxidant enzymes.
(3) It was concluded that supplementation of l-theanine up to 200 mg/kg can enhance meat quality (muscle color, pH and water holding capacity), anti-oxidant status, immune response, growth performance and lower total serum cholesterol in chickens.
(4) The level of l-theanine at 200 mg/kg of diet was found optimum and would be advised for getting better immune and performance responses in broilers. Higher dietary levels of l-theanine may pose deleterious effects on performance and other health aspects in birds.

## Figures and Tables

**Table 1:** Growth performance traits of broilers as affected by dietary l-theanine supplementation within the whole experimental period and the intervals.

Items	C	100LT	200LT	300LT	SEM	*p* Value
1–3 week of age						
BWG	922.85 ^b^	998.84 ^b^	1131.0 ^a^	905.78 ^b^	1.98	0.002
FC	1620.0 ^bc^	1566.0 ^c^	1757.0 ^a^	1690.1 ^ab^	1.51	0.015
FCR	1.7625 ^a^	1.5759 ^b^	1.5547 ^b^	1.8660 ^a^	0.47	0.002
3–6 week of age						
BWG	1423.2 ^c^	1511.4 ^bc^	1700.8 ^a^	1596.2 ^ab^	1.85	0.007
FC	2571.5 ^b^	2626.6 ^ab^	2750.0 ^a^	2680.0 ^ab^	1.74	0.121
FCR	1.8111 ^a^	1.7452 ^ab^	1.6179 ^b^	1.6842 ^ab^	0.51	0.185
1–6 week of age						
BWG	2546.1 ^c^	2710.3 ^b^	2931.8 ^a^	2702.0 ^b^	1.58	0.001
FC	4191.5 ^b^	4192.6 ^b^	4507.0 ^a^	4370.1 ^ab^	1.24	0.008
FCR	1.6462 ^a^	1.5469 ^bc^	1.5372 ^c^	1.6173 ^ab^	0.24	0.002

C: control (basal diet), 100LT: basal diet + 100 mg l-theanine/kg diet, 200LT: basal diet + 200 mg l-theanine/kg diet, 300LT: basal diet + 300 mg l-theanine/kg diet. BWG: body weight gain, FC: feed consumed, FCR: feed conversion ratio. Means in the same row with a common superscript letter following them are not significantly different (*p* ≤ 0.05).

**Table 2:** Carcass traits and weight of immune organs of broilers as affected by dietary l-theanine supplementation at 42 days of age.

Items (g)	C	100LT	200LT	300LT	SEM	*p* Value
Live weight	2525.0 ^d^	2810.0 ^b^	2925.0 ^a^	2650.0 ^c^	15.32	0.021
Slaughter weight	2478.8	2805.0	2760.0	2516.3	14.25	0.654
Semi-eviscerated weight	2350.0	2630.0	2611.3	2531.3	25.65	0.248
Eviscerated weight	2028.0 ^b^	2286.0 ^a^	2289.0 ^a^	2205.0 ^c^	16.35	0.010
Leg muscles	191.25	213.75	212.50	212.25	1.65	0.398
Breast muscles	306.25	343.75	368.75	337.50	4.35	0.214
Abdominal fat	67.60	70.44	70.80	64.43	1.35	0.895
Liver	35.23	36.07	35.21	35.10	2.35	0.542
Gizzard	16.63 ^b^	17.88 ^ab^	20.48 ^a^	17.10 ^b^	1.22	0.003
Heart	7.65	7.84	8.23	7.69	0.98	0.879
Thymus	1.77	2.72	2.06	2.39	0.42	0.845
Spleen	1.11 ^b^	1.37 ^ab^	1.66 ^a^	1.31 ^ab^	0.19	0.042
Bursa	1.32 ^c^	2.73 ^ab^	2.83 ^a^	1.98 ^bc^	0.15	0.021

C: control (basal diet), 100LT: basal diet + 100 mg l-theanine/kg diet, 200LT: basal diet + 200 mg l-theanine/kg diet, 300LT: basal diet + 300 mg l-theanine/kg diet. Means in the same row with a common superscript letter following them are not significantly different (*p* ≤ 0.05).

**Table 3:** Meat quality traits of broilers as affected by dietary l-theanine supplementation within 42 days of age.

Items (g)		C	100LT	200LT	300LT	SEM	*p* Value
BMC	L*	50.65 ^ab^	49.43 ^ab^	47.36 ^b^	52.59 ^a^	0.15	0.031
	a*	5.74 ^c^	7.35 ^b^	8.68 ^a^	6.22 ^b^	0.11	0.036
	b*	18.19 ^c^	19.92 ^ab^	19.00 ^bc^	21.25 ^a^	0.01	0.025
BM pH	45 min	6.89 ^a^	6.64 ^b^	6.64 ^b^	6.68 ^b^	0.02	0.040
	24 h	6.39	6.37	6.48	6.48	0.04	0.065
TMC	L*	65.48 ^a^	53.55 ^bc^	50.16 ^c^	58.79 ^b^	0.11	0.043
	a*	5.48 ^b^	6.39 ^ab^	6.94 ^a^	5.78 ^b^	0.05	0.036
	b*	18.56	19.39	20.26	19.86	0.51	0.276
TM pH	45 min	6.76 ^a^	6.49 ^c^	6.63 ^b^	6.63 ^b^	0.02	0.031
	24 h	6.96	6.81	6.75	6.72	0.37	0.152
WHC (breast)		0.33	0.44	0.46	0.47	0.25	0.055
WHC (thigh)		0.42 ^c^	0.67 ^a^	0.62 ^ab^	0.55 ^b^	0.09	0.045

C: control (basal diet), 100LT: basal diet + 100 mg l-theanine/kg diet, 200LT: basal diet + 200 mg l-theanine/kg diet, 300LT: basal diet+ 300 mg l-theanine/kg diet. BMC: breast meat color, L*: lightness, a*, redness, b*: yellowness, BM pH: breast meat pH, TMC: thigh meat color, TM pH: thigh meat pH, WHC: water-holding capacity. Means in the same row with a common superscript letter following them are not significantly different (*p* ≤ 0.05).

**Table 4:** Effect of l-theanine on villus height and crypt depth.

Items	Group	Villus Height (μm)	Crypt Depth (μm)	Villus Height/Crypt Depth
Jejunum	Control	1212.27 ± 47.79 ^a^	249.78 ± 12.50 ^a^	4.86 ± 0.29
	200 mg/kg	1381.83 ± 36.21 ^b^	370.41 ± 10.03 ^b^	3.69 ± 0.37
Ileum	Control	1175.01 ± 59.35 ^a^	213.96 ± 33.02 ^a^	5.58 ± 0.56
	200 mg/kg	1417.10 ± 75.12 ^b^	411.05 ± 45.39 ^b^	3.49 ± 0.51

Means in the same column with a different superscript letter following them are significantly different (*p* ≤ 0.05).

**Table 5:** Effect of l-theanine on histomorphometry of pectoral and gastrocnemius muscles.

Items	Group	Mean Fibre Cross-Sectional Area (μm)
Gastrocnemius	Control	107.82
	200 mg/kg	109.51
Pectoral	Control	99.37
	200 mg/kg	99.41

Means in the same column with no superscript letter following them are not significantly different (*p* ≤ 0.05).

**Table 6:** Blood chemistry of broilers as affected by dietary l-theanine supplementation within 42 days of age.

Items (g)	C	100LT	200LT	300LT	SEM	*p* Value
ALT (U/L)	4.38	3.92	3.75	4.27	0.85	0.658
AST (U/L)	238.15	234.28	205.90	236.5	4.56	0.956
T-chol (mmol/L)	2.54 ^a^	2.39 ^ab^	1.53 ^b^	2.45 ^ab^	0.45	0.020
TG (mmol/L)	0.38	0.320	0.37	0.40	0.02	1.325
HDL-c (mmol/L)	1.75	2.07	2.24	1.74	0.21	0.566
LDL-c (mmol/L)	0.54	0.51	0.45	0.63	0.03	0.523

C: control (basal diet), 100LT: basal diet + 100 mg l-theanine/kg diet, 200LT: basal diet + 200 mg l-theanine/kg diet, 300LT: basal diet + 300 mg l-theanine/kg diet. ALT: Alanine amino transferase, AST: Aspartate amino transferase, T-chol: total cholesterol, HDL-c: high density lipoprotein cholesterol, LDL-c: low density lipoprotein cholesterol. Means in the same row with no superscript letters after them or with a common superscript letter following them are not significantly different (*p* ≤ 0.05).

**Table 7:** Composition and chemical analysis of the basal diets.

Ingredients g/kg	Basal Diets	
	Starter (1–3 Weeks)	Finisher (3–6 Weeks)
Yellow Corn	571.3	605.3
Soybean meal	316.5	271.5
Gluten meal	65	61
Di Calcium phosphate	17	15
Limestone	12.4	11.5
Vitamin Premix *	3	3
NaCl	3	3
DL Methionine	0.5	0.2
l-Lysine	1.3	10
Soybean oil	10	19.5
Total	1000 g	1000 g
Calculated analysis **		
CP g/kg	230	210
ME Kcal/kg diet	2951	3099
Ca g/kg	10	9
P (Available) g/kg	4.5	4
Lysine g/kg	12	10.5
M+C g/kg	8.3	7.4
CF g/kg	35.6	33.1

* Growth vitamin and Mineral premix Each 2.5 kg consists of: Vit. A 12,000, 000 IU; Vit. D3, 2000, 000 IU; Vit. E. 10 g; Vit. K 32 g; Vit. B1, 1000 mg; Vit. B2, 49 g; Vit. B6, 105 g; Vit. B12, 10 mg; Pantothenic acid, 10 g; Niacin, 20 g, Folic acid, 1000 mg; Biotin, 50 g; Choline Chloride, 500 mg, Fe, 30 g; Mn, 40 g; Cu, 3 g; Co., 200 mg; Si, 100 mg and Zn, 45 g. ** Calculated according to NRC (1994).

**Table 8:** List of specific primers sequence were used for *IL-6*, *IFN-γ*, *IL-2*, and *TNF-α* cytokine gene expression.

Gene	Nucleotide Sequence (5′-3′)	Amplification of Length
*IL-6*	Forward primer: TGGTGATAAATCCCGATGAAG Reverse primer: GGCACTGAAACTCCTGGTCT	191
*IFN-γ*	Forward primer: ATCATACTGAGCCAGATTGTTTCG Reverse primer: TCTTTCACCTTCTTCACGCCAT	140
*IL-2*	Forward primer: GCTAATGACTACAGCTTATGGAGCA Reverse primer: TGGGTCTCAGTTGGTGTGTAGAG	135
*TNF-α*	Forward primer: AGATGGGAAGGGAATGAACC Reverse primer: CAGAGCATCAACGCAAAAG	268

## References

[1] Bhatti M. (2011). Emerging prospects of poultry production in Pakistan at the dawn of 21st century. Vet. News Views.

[2] Dhama K., Latheef S.K., Mani S., Samad H.A., Karthik K., Tiwari R., Khan R.U., Alagawany M., Farag M.R., Alam G.M. (2015). Multiple beneficial applications and modes of action of herbs in poultry health and production—A review. Int. J. Pharmacol..

[3] Saeed M., Baloch A.R., Wang M., Soomro R.N., Baloch A.M., Bux B.A., Arian M.A., Faraz S.S., Zakriya H.M. (2015). Use of *Cichorium intybus* Leaf extracts as a growth promoter, hepatoprotectant and immune modulent in broilers. J. Anim. Prod. Adv..

[4] Ayssiwede S., Dieng A., Bello H., Chrysostome C., Hane M., Mankor A., Dahouda M., Houinato M., Hornick J., Missohou A. (2011). Effects of *Moringa oleifera* (Lam.) leaves meal incorporation in diets on growth performances, carcass characteristics and economics results of growing indigenous senegal chicken. Pak. J. Nutr..

[5] El-Hack M.E.A., Alagawany M., Farag M.R., Tiwari R., Karthik K., Dhama K. (2016). Nutritional, healthical and therapeutic efficacy of black cumin (*Nigella sativa*) in animals, poultry and humans. Int. J. Pharmacol..

[6] Al-Basher G., Ajarem J.S., Allam A.A., Mahmoud A.M. (2017). Green tea protects against perinatal nicotine-induced histological, biochemical and hematological alterations in mice off spring. Int. J. Pharmacol..

[7] Lin X., Lin C.-H., Zhao T., Zuo D., Ye Z., Liu L., Lin M.T. (2017). Quercetin protects against heat stroke-induced myocardial injury in male rats: Antioxidative and antiinflammatory mechanisms. Chem. Biol. Interact..

[8] Akinloye O.A., Somade O.T., Akindele A.S., Adelabu K.B., Elijah F.T., Adewumi O.J. (2014). Anticlastogenic and hepatoprotective properties of ginger (*Zingiber officinale*) extract against nitrobenzene-induced toxicity in rats. Roman. J. Biochem..

[9] Alagawany M., El-Hack M.E.A., El-Kholy M.S. (2016). Productive performance, egg quality, blood constituents, immune functions, and antioxidant parameters in laying hens fed diets with different levels of *Yucca schidigera* extract. Environ. Sci. Pollut. Res..

[10] Saeed M., Abd El-Hack M., Alagawany M., Arain M., Arif M., Mirza M., Naveed M., Chao S., Sarwar M., Sayab M. (2017). Nutritional and Healthical Aspects of Yacon (*Smallanthus sonchifolius*) for Human, Animals and Poultry. Int. J. Pharmacol..

[11] Chacko S.M., Thambi P.T., Kuttan R., Nishigaki I. (2010). Beneficial effects of green tea: A literature review. Chin. Med..

[12] Abdo Z.M., Hassan R., El-Salam A.A., Helmy S.A. (2010). Effect of adding green tea and its aqueous extract as natural antioxidants to laying hen diet on productive, reproductive performance and egg quality during storage and its content of cholesterol. Egypt. Poult. Sci. J..

[13] Kojima S., Yoshida Y. (2008). Effects of green tea powder feed supplement on performance of hens in the late stage of laying. Int. J. Poult. Sci..

[14] Eschenauer G., Sweet B.V. (2006). Pharmacology and therapeutic uses of theanine. Am. J. Health Syst. Pharm..

[15] Deng W.W., Ogita S., Ashihara H. (2010). Distribution and biosynthesis of theanine in Theaceae plants. Plant Physiol. Biochem..

[16] Tian X., Sun L., Gou L., Ling X., Feng Y., Wang L., Yin X., Liu Y. (2013). Protective effect of l-theanine on chronic restraint stress-induced cognitive impairments in mice. Brain Res..

[17] Kurihara S., Shibahara S., Arisaka H., Akiyama Y. (2007). Enhancement of antigen-specific immunoglobulin G production in mice by co-administration of l-cystine and l-theanine. Vet. Med. Sci..

[18] Yang H., Li W., Yu H., Yuan R., Yang Y., Pung K., Li P., Xue L. (2013). Physiological effects of l-Theanine on *Drosophila melanogaster*. Molecules.

[19] Li C., Tong H., Yan Q., Tang S., Han X., Xiao W., Tan Z. (2016). l-Theanine Improves Immunity by Altering TH2/TH1 Cytokine Balance, Brain Neurotransmitters, and Expression of Phospholipase C in Rat Hearts. Med. Sci. Monit. Int. Med. J. Exp. Clin. Res..

[20] Yin C., Gou L., Liu Y., Yin X., Zhang L., Jia G., Zhuang X. (2011). Antidepressant-like effects of l-theanine in the forced swim and tail suspension tests in mice. Phytother. Res..

[21] Takeda A., Tamano H., Suzuki M., Sakamoto K., Oku N., Yokogoshi H. (2012). Unique induction of CA1 LTP components after intake of theanine, an amino acid in tea leaves and its effect on stress response. Cell. Mol. Neurobiol..

[22] Wen H., Wei S., Zhang S., Hou D., Xiao W., He X. (2012). Effects of l-theanine on performance and immune function of yellow-feathered broilers. Chin. J. Anim. Nutr..

[23] Hwang Y., Park B., Lim J., Kim M., Song I., Park S., Jung H., Hong J., Yun H. (2008). Effects of β-Glucan from *Paenibacillus polymyxa* and l-theanine on Growth Performance and Immunomodulation in Weanling Piglets. Asian-Australas. J. Anim. Sci..

[24] Williams J., Kellett J., Roach P.D., Mckune A., Mellor D., Thomas J., Naumovski N. (2016). l-theanine as a functional food additive: Its role in disease prevention and health promotion. Beverages.

[25] Biswas A.H., Wakita M. (2001). Effect of dietary Japanese green tea powder supplementation on feed utilization and carcass profiles in broilers. J. Poult. Sci..

[26] Sarker M., Kim G., Yang C. (2010). Effect of green tea and biotite on performance, meat quality and organ development in Ross broiler. Egypt. Poult. Sci. J..

[27] Erener G., Ocak N., Altop A., Cankaya S., Aksoy H.M., Ozturk E. (2011). Growth performance, meat quality and caecal coliform bacteria count of broiler chicks fed diet with green tea extract. Asian-Australas. J. Anim. Sci..

[28] Uuganbayar D. (2004). A Study on the Utilization of Green Tea for Laying Hens and Broiler Chicks. Ph.D. Thesis.

[29] Yang C., Yang I., Oh D., Bae I., Cho S., Kong I., Uuganbayar D., Nou I., Choi K. (2003). Effect of green tea by-product on performance and body composition in broiler chicks. Asian-Australas. J. Anim. Sci..

[30] Kamath A.B., Wang L., Das H., Li L., Reinhold V.N., Bukowski J.F. (2003). Antigens in tea-beverage prime human Vγ2Vδ2 T cells in vitro and in vivo for memory and nonmemory antibacterial cytokine responses. Proc. Natl. Acad. Sci. USA.

[31] Afsharmanesh M., Sadaghi B. (2014). Effects of dietary alternatives (probiotic, green tea powder, and Kombucha tea) as antimicrobial growth promoters on growth, ileal nutrient digestibility, blood parameters, and immune response of broiler chickens. Comp. Clin. Pathol..

[32] Bukowski J.F., Morita C.T., Brenner M.B. (1999). Human cd T cells recognise alkylamines derived from microbes, edible plants, tea: Implications for innate immunity. Immunity.

[33] Zheng G., Sayama K., Okubo T., Juneja L.R., Oguni I. (2004). Anti-obesity effects of three major components of green tea, catechins, caffeine and theanine, in mice. In Vivo.

[34] Bukowski J.F., Percival S.S. (2008). l-theanine intervention enhances human γδ T lymphocyte function. Nutr. Rev..

[35] Matsumoto K., Yamada H., Takuma N., Niino H., Sagesaka Y.M. (2011). Effects of green tea catechins and theanine on preventing influenza infection among healthcare workers: A randomized controlled trial. BMC Complement. Altern. Med..

[36] Kurihara S., Hiraoka T., Akutsu M., Sukegawa E., Bannai M., Shibahara S. (2010). Effects of l-cystine and l-theanine supplementation on the common cold: A randomized, double-blind, and placebo-controlled trial. J. Amin. Acids.

[37] Takagi Y., Kurihara S., Higashi N., Morikawa S., Kase T., Maeda A., Arisaka H., Shibahara S., Akiyama Y. (2010). Combined administration of l-cystine and l-theanine enhances immune functions and protects against influenza virus infection in aged mice. J. Vet. Med. Sci..

[38] Fletcher D. (1999). Broiler breast meat color variation, pH, and texture. Poult. Sci..

[39] Qiao M., Fletcher D., Smith D., Northcutt J. (2001). The effect of broiler breast meat color on pH, moisture, water-holding capacity, and emulsification capacity. Poult. Sci..

[40] Kim Y.H., Huff-Lonergan E., Sebranek J.G., Lonergan S.M. (2010). High-oxygen modified atmosphere packaging system induces lipid and myoglobin oxidation and protein polymerization. Meat Sci..

[41] Young J.F., Stagsted J., Jensen S.K., Karlsson A., Henckel P. (2003). Ascorbic acid, alpha-tocopherol, and oregano supplements reduce stress-induced deterioration of chicken meat quality. Poult. Sci..

[42] Yan Q., Tong H., Tang S., Tan Z., Han X., Zhou C. (2017). l-theanine administration modulates the absorption of dietary nutrients and expression of transporters and receptors in the intestinal mucosa of rats. BioMed Res. Int..

[43] Pelícia K., Mendes A., Saldanha E., Pizzolante C., Takahashi S., Garcia R., Moreira J., Paz I., Quinteiro R., Komiyama C. (2004). Probiotic and prebiotic utilization in diets for free-range broiler chickens. Revista Brasileira de Ciência Avícola.

[44] Mosleh N., Shomali T., Hamedi S. (2011). Effects of green tea on performance, feed conversion and jejunum (histology) in broilers. Online J. Vet. Res..

[45] Pluske J.R., Thompson M.J., Atwood C.S., Bird P.H., Williams I.H., Hartmann P.E. (1996). Maintenance of villus height and crypt depth, and enhancement of disaccharide digestion and monosaccharide absorption, in piglets fed on cows’ whole milk after weaning. Br. J. Nutr..

[46] Abdelqader A., Al-Fataftah A.R. (2016). Effect of dietary butyric acid on performance, intestinal morphology, microflora composition and intestinal recovery of heat-stressed broilers. Livest. Sci..

[47] Zhao D. (2002). Review on research status of theanine. Food Sci..

[48] Murakami S., Kurihara S., Titchenal C.A., Ohtani M. (2010). Suppression of exercise-induced neutrophilia and lymphopenia in athletes by cystine/theanine intake: A randomized, double-blind, placebo-controlled trial. J. Int. Soc. Sports Nutr..

[49] Ariana M., Samie A., Edriss M.A., Jahanian R. (2011). Effects of powder and extract form of green tea and marigold, and-tocopheryl acetate on performance, egg quality and egg yolk cholesterol levels of laying hens in late phase of production. J. Med. Plant Res..

[50] Abdel-Azeem F. (2005). Green tea flowers (*Camellia sinensis*) as natural anti-oxidants feed additives in growing Japanese quail diets. Egypt. Poult. Sci. J..

[51] Raederstorff D.G., Schlachter M.F., Elste V., Weber P. (2003). Effect of EGCG on lipid absorption and plasma lipid levels in rats. J. Nutr. Biochem..

[52] Zheng G., Bamba K., Okubo T., Raj Juneja L., Oguni I., Sayama K. (2005). Effect of theanine, γ-glutamylethylamide, on bodyweight and fat accumulation in mice. Anim. Sci. J..

[53] El-Deek A., Al-Harthi M. (2004). Responses of modern broiler chicks to stocking density, green tea, commercial multi-enzymes and their interactions on productive performance, carcass characteristics, liver composition and plasma constituents. Int. J. Poult. Sci..

[54] Xu X., Hu Y., Xiao W., Huang J.A., He X., Wu J., Ryan E.P., Weir T.L. (2012). Effects of fermented *Camilla sinensis*, Fuzhuan tea, on egg cholesterol and production performance in laying hens. Agric. Food Sci..

[55] Ahmad R.S., Butt M.S., Sultan M.T., Mushtaq Z., Ahmad S., Dewanjee S., de Feo V., Zia-Ul-Haq M. (2015). Preventive role of green tea catechins from obesity and related disorders especially hypercholesterolemia and hyperglycemia. J. Transl. Med..

[56] Yousaf S., Butt M., Suleria H., Iqbal M. (2014). The role of green tea extract and powder in mitigating metabolic syndromes with special reference to hyperglycemia and hypercholesterolemia. Food Funct..

[57] Koo S.I., Sang K.N. (2007). Green tea as inhibitor of the intestinal absorption of lipids: Potential mechanism for its lipid-lowering effect. J. Nutr. Biochem..

[58] Tayal V., Kalra B.S. (2008). Cytokines and anti-cytokines as therapeutics—An update. Eur. J. Pharmacol..

[59] Li G., Ye Y., Kang J., Yao X., Zhang Y., Jiang W., Gao M., Dai Y., Xin Y., Wang Q. (2012). l-Theanine prevents alcoholic liver injury through enhancing the antioxidant capability of hepatocytes. Food Chem. Toxicol..

[60] Jiang W., Gao M., Sun S., Bi A., Xin Y., Han X. (2012). Protective effect of l-theanine on carbon tetrachloride-induced acute liver injury in mice. Biochem. Biophys. Res. Commun..

[61] Pi Z., Wu Y., Liu J. (2005). Effect of pretreatment and pelletization on nutritive value of rice straw-based total mixed ration, and growth performance and meat quality of growing Boer goats fed on TMR. Small Rumin. Res..

[62] Liu Z., Gu H., Gan L., Xu Y., Feng F., Saeed M., Sun C. (2017). Reducing Smad3/ATF4 was essential for Sirt1 inhibiting ER stress-induced apoptosis in mice brown adipose tissue. Oncotarget.

[63] Sikandar A., Cheema A., Younus M., Aslam A., Zaman M., Rehman T. (2012). Histopathological and serological studies on paratuberculosis in cattle and buffaloes. Pak. Vet. J..

[64] Schneider C.A., Rasband W.S., Eliceiri K.W. (2012). Nih image to ImageJ: 25 Years of image analysis. Nat. Methods.

[65] Ashraf S., Zaneb H., Yousaf M.S., Ijaz A., Sohail M.U., Muti S. (2013). Effect of dietary supplementation of prebiotics and probiotics on intestinal microarchitecture in broilers reared under cyclic heat stress. J. Anim. Physiol. Anim. Nutr..

[66] Kiczorowska B., Al-Yasiry A., Samolińska W., Marek A., Pyzik E. (2016). The effect of dietary supplementation of the broiler chicken diet with Boswellia serrata resin on growth performance, digestibility, and gastrointestinal characteristics, morphology, and microbiota. Livest. Sci..

[67] Heywood J., Mcentee G., Stickland N. (2005). In ovo neuromuscular stimulation alters the skeletal muscle phenotype of the chick. J. Muscle Res. Cell Motil..

